# Personality traits, emotional intelligence and decision-making styles in Lebanese universities medical students

**DOI:** 10.1186/s40359-020-00406-4

**Published:** 2020-05-05

**Authors:** Radwan El Othman, Rola El Othman, Rabih Hallit, Sahar Obeid, Souheil Hallit

**Affiliations:** 1 Faculty of Medicine and Medical Sciences, Holy Spirit University of Kaslik (USEK), Jounieh, Lebanon; 2 Department of Pediatrics, Bahman Hospital, Beirut, Lebanon; 3 Department of Infectious Disease, Bellevue Medical Center, Mansourieh, Lebanon; 4 Department of Infectious Disease, Notre Dame des Secours University Hospital Center, Byblos, Lebanon; 5 Research and Psychology departments, Psychiatric Hospital of the Cross, P.O. Box 60096, Jal Eddib, Lebanon; 6 Faculty of Arts and Sciences, Holy Spirit University of Kaslik (USEK), Jounieh, Lebanon; 7 INSPECT-LB: Institut National de Santé Publique, Epidémiologie Clinique et Toxicologie – Liban, Beirut, Lebanon

**Keywords:** Personality traits, Big five, Decision-making, Decision-making style, Emotional intelligence, Medical students

## Abstract

**Background:**

This study aims to assess the impact of personality traits on emotional intelligence (EI) and decision-making among medical students in Lebanese Universities and to evaluate the potential mediating role-played by emotional intelligence between personality traits and decision-making styles in this population.

**Methods:**

This cross-sectional study was conducted between June and December 2019 on 296 general medicine students.

**Results:**

Higher extroversion was associated with lower rational decision-making style, whereas higher agreeableness and conscientiousness were significantly associated with a higher rational decision-making style. More extroversion and openness to experience were significantly associated with a higher intuitive style, whereas higher agreeableness and conscientiousness were significantly associated with lower intuitive style. More agreeableness and conscientiousness were significantly associated with a higher dependent decision-making style, whereas more openness to experience was significantly associated with less dependent decision-making style. More agreeableness, conscientiousness, and neuroticism were significantly associated with less spontaneous decision-making style. None of the personality traits was significantly associated with the avoidant decision-making style. Emotional intelligence seemed to fully mediate the association between conscientiousness and intuitive decision-making style by 38% and partially mediate the association between extroversion and openness to experience with intuitive decision-making style by 49.82 and 57.93% respectively.

**Conclusion:**

Our study suggests an association between personality traits and decision-making styles. The results suggest that EI showed a significant positive effect on intuitive decision-making style and a negative effect on avoidant and dependent decision-making styles. Additionally, our study underlined the role of emotional intelligence as a mediator factor between personality traits (namely conscientiousness, openness, and extroversion) and decision-making styles.

## Background

Decision-making is a central part of daily interactions; it was defined by Scott and Bruce in 1995 as «the learned habitual response pattern exhibited by an individual when confronted with a decision situation. It is not a personality trait, but a habit-based propensity to react in a certain way in a specific decision context» [1]. Understanding how people make decisions within the moral domain is of great importance theoretically and practically. Its theoretical value is related to the importance of understanding the moral mind to further deepen our knowledge on how the mind works, thus understanding the role of moral considerations in our cognitive life. Practically, this understanding is important because we are highly influenced by the moral decisions of people around us [2]. According to Scott and Bruce (1995), there are five distinct decision-making styles (dependent, avoidant, spontaneous, rational, intuitive) [1] and each individuals’ decision-making style has traits from these different styles with one dominant style [3].

The dependent decision-making style can be regarded as requiring support, advice, and guidance from others when making decisions. Avoidant style is characterized by its tendency to procrastinate and postpone decisions if possible. On the other hand, spontaneous decision-making style is hallmarked by making snap and impulsive decisions as a way to quickly bypass the decision-making process. In other words, spontaneous decision-makers are characterized by the feeling of immediacy favoring to bypass the decision-making process rapidly without employing much effort in considering their options analytically or relying on their instinct. Rational decision-making style is characterized by the use of a structured rational approach to analyze information and options to make decision [1]. In contrast, intuitive style is highly dependent upon premonitions, instinct, and feelings when it comes to making decisions driving focus toward the flow of information rather than systematic procession and analysis of information, thus relying on hunches and gut feelings. Several studies have evaluated the factors that would influence an individual’s intuition and judgment. Rand et al. (2016) discussed the social heuristics theory and showed that women and not men tend to internalize altruism _ the selfless concern for the well-being of others_ in their intuition and thus in their intuitive decision-making process [4]. Additionally, intuitive behavior honesty is influenced by the degree of social relationships with individuals affected by the outcome of our decision: when dishonesty harms abstract others, intuition promotion causes more dishonesty. On the contrary, when dishonesty harms concrete others, intuition promotion has no significant effect on dishonesty. Hence, the intuitive appeal of pro-sociality may cancel out the intuitive selfish appeal of dishonesty [5]. Moreover, the decision-making process and styles have been largely evaluated in previous literature. Greene et al. (2008) and Rand (2016) showed that utilitarian moral judgments aiming to minimize cost and maximize benefits across concerned individuals are driven by controlled cognitive process (i.e. rational); whereas, deontological moral judgments _where rights and duties supersede utilitarian considerations_ are dictated by an automatic emotional response (e.g. spontaneous decision-making) [6, 7]. Trémolière et al. (2012) found that mortality salience makes people less utilitarian [8].

Another valuable element influencing our relationships and career success [9] is emotional intelligence (EI) a cardinal factor to positive patient experience in the medical field [10]. EI was defined by Goleman as «the capacity of recognizing our feelings and those of others, for motivating ourselves, and for managing emotions both in us and in our relationships» [11]. Hence, an important part of our success in life nowadays is dependent on our ability to develop and preserve social relationships, depict ourselves positively, and control the way people descry us rather than our cognitive abilities and traditional intelligence measured by IQ tests [12]. In other words, emotional intelligence is a subtype of social intelligence involving observation and analyses of emotions to guide thoughts and actions. Communication is a pillar of modern medicine; thus, emotional intelligence should be a cornerstone in the education and evaluation of medical students’ communication and interpersonal skills.

An important predictor of EI is personality [13] defined as individual differences in characteristic patterns of thinking, feeling and behaving [14]. An important property of personality traits is being stable across time [15] and situations [16], which makes it characteristic of each individual. One of the most widely used assessment tools for personality traits is the Five-Factor model referring to «extroversion, openness to experience, agreeableness, conscientiousness, neuroticism». In fact, personality traits have an important impact on individuals’ life, students’ academic performance [17] and decision-making [18].

Extroversion is characterized by higher levels of self-confidence, positive emotions, enthusiasm, energy, excitement seeking, and social interactions. Openness to experience individuals are creative, imaginative, intellectually curious, impulsive, and original, open to new experiences and ideas [19]. Agreeableness is characterized by cooperation, morality, sympathy, low self-confidence, high levels of trust in others, and tend to be happy and satisfied because of their close interrelationships [19]. Conscientiousness is characterized by competence, hard work, self-discipline, organization, strive for achievement and goal orientation [20] with a high level of deliberation making conscientious individuals capable of analyzing the pros and cons of a given situation [21]. Neuroticism is characterized by anxiety, anger, insecurity, impulsiveness, self-consciousness,and vulnerability [20]. High neurotic individuals have higher levels of negative affect, are easily irritated, and more likely to turn to inappropriate coping responses, such as interpersonal hostility [22].

**Fig. 1:**
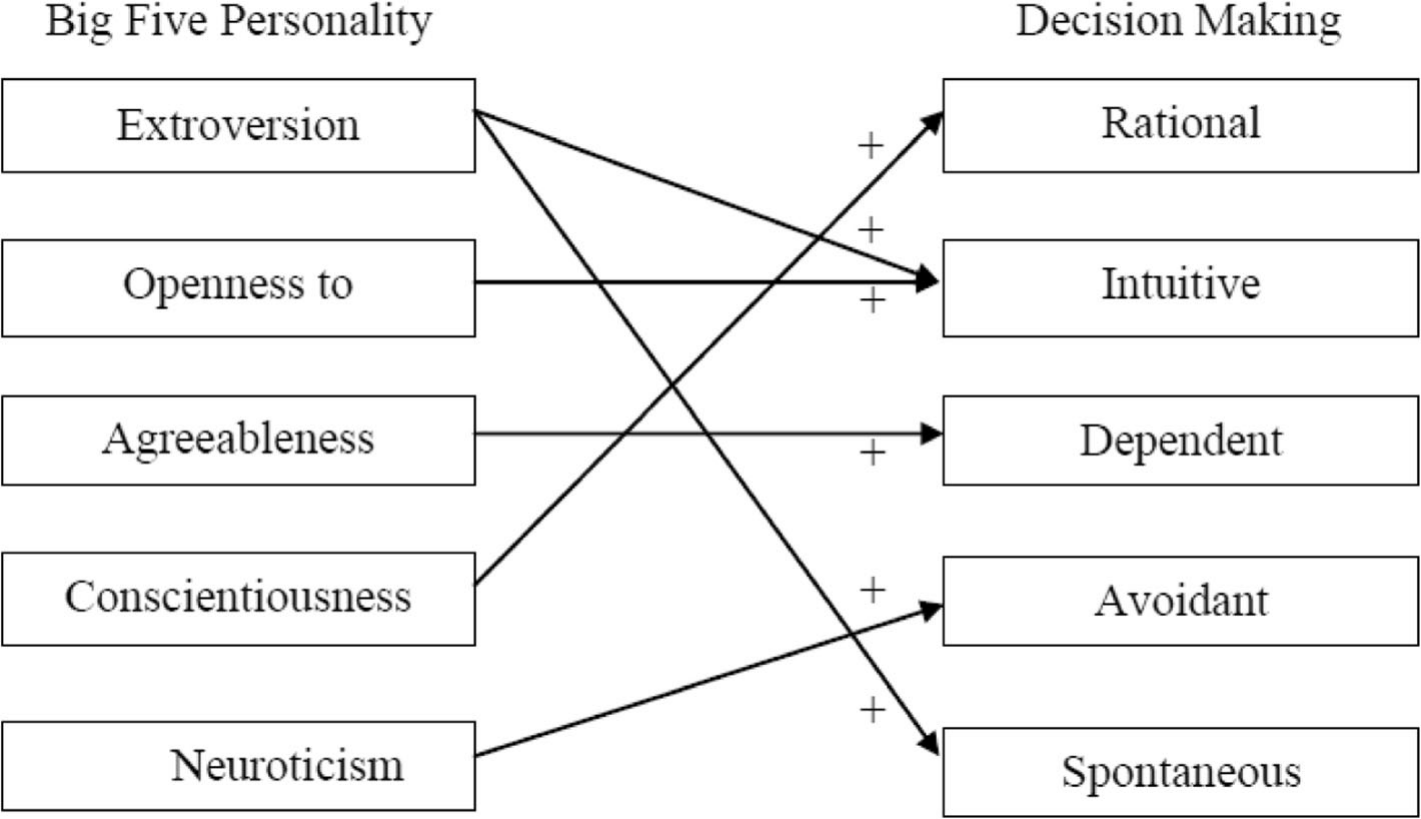
Schematic representation of the effect of the big five personality types on decision-making styles [24]

Multiple studies have evaluated the impact of personality traits on decision-making styles. Narooi and Karazee (2015) studied personality traits, attitude to life, and decision-making styles among university students in Iran [23]. They deduced the presence of a strong relationship between personality traits and decision-making styles [23]. Riaz and Batool (2012) evaluated the relationship between personality traits and decision-making among a group of university students (Fig. 1). They concluded that «15.4 to 28.1% variance in decision-making styles is related to personality traits» [24]. Similarly, Bajwa et al. (2016) studied the relationship between personality traits and decision-making among students. They concluded that conscientiousness personality trait is associated with rational decision-making style [25]. Bayram and Aydemir (2017) studied the relationship between personality traits and decision-making styles among a group of university students in Turkey [26]. Their work yielded to multiple conclusion namely a significant association between rational and intuitive decision-making styles and extroversion, openness to experience, conscientiousness, and agreeableness personality traits [26]. The dependent decision-making style had a positive relation with both neuroticism and agreeableness. The spontaneous style had a positive relation with neuroticism and significant negative relation with agreeableness and conscientiousness. Extroversion personality traits had a positive effect on spontaneous style. Agreeableness personality had a positive effect on the intuitive and dependent decision-making style. Conscientiousness personality had a negative effect on avoidant and spontaneous decision-making style and a positive effect on rational style. Neuroticism trait had a positive effect on intuitive, dependent and spontaneous decision-making style. Openness to experience personality traits had a positive effect on rational style [26].

Furthermore, several studies have evaluated the relationship between personality traits and emotional intelligence. Dawda and Hart (2000) found a significant relationship between emotional intelligence and all Big Five personality traits [27]. Day and al. (2005) found a high correlation between emotional intelligence and extroversion and conscientiousness personality traits [28]. A study realized by Avsec and al. (2009) revealed that emotional intelligence is a predictor of the Big Five personality traits [29]. Alghamdi and al. (2017) investigated the predictive role of EI on personality traits among university advisors in Saudi Arabia. They found that extroversion, agreeableness, and openness to experience emerged as significant predictors of EI. The study also concluded that conscientiousness and neuroticism have no impact on EI [13].

Nonetheless, decision-making is highly influenced by emotion making it an emotional process. The degree of emotional involvement in a decision may influence our choices [30] especially that emotions serve as a motivational process for decision-making [31]. For instance, patients suffering from bilateral lesions of the ventromedial prefrontal cortex (interfering with normal processing of emotional signals) develop severe impairments in personal and social decision-making despite normal cognitive capabilities (intelligence and creativity); highlighting the guidance role played by emotions in the decision-making process [32]. Furthermore, EI affects attention, memory, and cognitive intelligence [33, 34] with higher levels of EI indicating a more efficient decision-making [33]. In one study, Khan and al. concluded that EI had a significant positive effect on rational and intuitive decision-making styles and negative effect on dependent and spontaneous decision-making styles among a group of university students in Pakistan [35].

This study aims to assess the impact of personality traits on both emotional intelligence and decision-making among medical students in Lebanese Universities and to test the potential mediating role played by emotional intelligence between personality and decision-making styles in this yet unstudied population to our knowledge. The goal of the present research is to evaluate the usefulness of implementing such tools in the selection process of future physicians. It also aimed at assessing the need for developing targeted measures, aiming to ameliorate the psychosocial profile of Lebanese medical students, in order to have a positive impact on patients experience and on medical students’ career success.

## Methods

### Study design

This cross-sectional study was conducted between June and December 2019. A total of 296 participants were recruited from all the 7 faculties of medicine in Lebanon. Data collection was done through filling an anonymous online or paper-based self-administered English questionnaire upon the participant choice. All participants were aware of the purpose of the study, the quality of data collected and gave prior informed consent. Participation in this study was voluntary and no incentive was given to the participants. All participants were General medicine students registered as full-time students in one of the 7 national schools of medicine aged 18 years and above regardless of their nationality. The questionnaire was only available in English since the 7 faculties of medicine in Lebanon require a minimum level of good English knowledge in their admission criteria. A pilot test was conducted on 15 students to check the clarity of the questionnaire. To note that these 15 questionnaires related data was not entered in the final database. The methodology used in similar to the one used in a previous paper [36]

### Questionnaire and variables

The questionnaire assessed demographic and health characteristics of participants, including age, gender, region, university, current year in medical education, academic performance (assessed using the current cumulative GPA), parental highest level of education, and health questions regarding the personal history of somatic, and psychiatric illnesses.

The personality traits were evaluated using the Big Five Personality Test, a commonly used test in clinical psychology. Since its creation by John, Donahue, and Kentle (1991) [37], the five factor model was widely used in different countries including Lebanon [38]; it describes personality in terms of five board factors: extroversion, openness to experience, agreeableness, conscientiousness and neuroticism according to an individual’s response to a set of 50 questions on a 5-point Likert scale: 1 (disagree) to 5 (agree). A score for each personality trait is calculated in order to determine the major trait(s) in an individual personality (i.e. the trait with the highest score). The Cronbach’s alpha values were as follows: total scale (0.885), extroversion (0.880), openness to experience (0.718), agreeableness (0.668), conscientiousness (0.640), and neuroticism (0.761).

Emotional intelligence was assessed using the Quick Emotional Intelligence Self-Assessment scale [38]. The scale is divided into four domains: «emotional alertness, emotional control, social-emotional awareness, and relationship management». Each domain is composed of 10 questions, with answers measured on a 5-point Likert scale: 0 (never) to 4 (always). Higher scores indicate higher emotional intelligence [38] (α_Cronbach_ = 0.950).

The decision-making style was assessed using the Scott and Bruce General Decision-Making Style Inventory commonly used worldwide since its creation in 1995 for this purpose [1]. The inventory consists of 25 questions answered according to a 5-point Likert scale: 1 (strongly disagree) to 5 (strongly agree) intended to evaluate the importance of each decision-making style among the 5 styles proposed by Scott and Bruce: dependent, avoidant, spontaneous, rational and intuitive. The score for each decision-making style is computed in order to determine the major style for each responder (α_Cronbach total scale_ = 0.744; α_Cronbach dependent style_ = 0.925; α_Cronbach avoidant style_ = 0.927; α_Cronbach spontaneous style_ = 0.935; α_Cronbach rational style_ = 0.933; α_Cronbach intuitive style_ = 0.919).

### Sample size calculation

The Epi info program (Centers for Disease Control and Prevention (CDC), Epi Info™) was employed for the calculation of the minimal sample size needed for our study, with an acceptable margin of error of 5% and an expected variance of decision-making styles that is related to personality types estimated by 15.4 to 28.1% [24] for 5531 general medicine student in Lebanon [39]. The result showed that 294 participants are needed.

### Statistical analysis

Statistical Package for Social Science (SPSS) version 23 was used for the statistical analysis. The Student *t-test* and ANOVA test were used to assess the association between each continuous independent variable (decision-making style scores) and dichotomous and categorical variables respectively. The Pearson correlation test was used to evaluate the association between two continuous variables. Reliability of all scales and subscales was assessed using Cronbach’s alpha.

### Mediation analysis

**Fig. 2:**
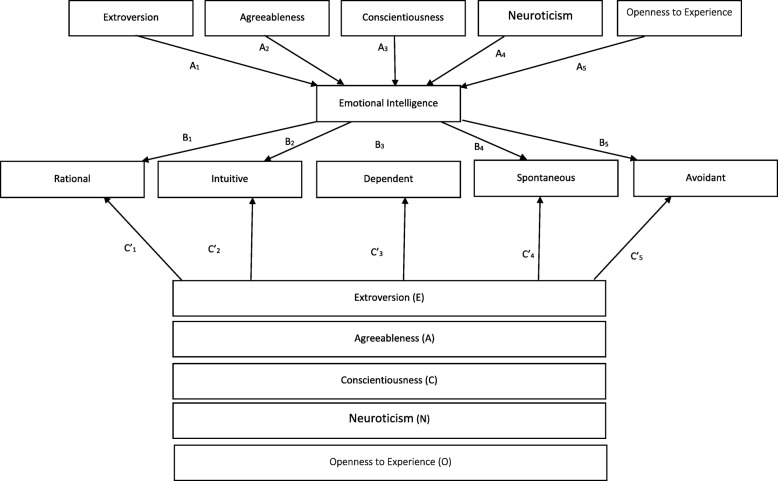
Summary of the pathways followed during the mediation analysis

The PROCESS SPSS Macro version 3.4, model four [40] was used to calculate five pathways (Fig. 2). Pathway A determined the regression coefficient for the effect of each personality trait on emotional intelligence, Pathway B examined the association between EI and each decision-making style, independent of the personality trait, and Pathway C′ estimated the total and direct effect of each personality trait on each decision-making style respectively. Pathway AB calculated the indirect intervention effects. To test the significance of the indirect effect, the macro generated bias-corrected bootstrapped 95% confidence intervals (CI) [40]. A significant mediation was determined if the CI around the indirect effect did not include zero [40]. The covariates that were included in the mediation model were those that showed significant associations with each decision-making style in the bivariate analysis.

## Results

### Sociodemographic and other characteristics of the participants

The mean age of the participants was 22.41 ± 2.20 years, with 166 (56.1%) females. The mean scores of the scales used were as follows: emotional intelligence (108.27 ± 24.90), decision-making: rationale style (13.07 ± 3.17), intuitive style (16.04 ± 3.94), dependent style (15.53 ± 4.26), spontaneous style (13.52 ± 4.22), avoidant style (12.44 ± 4.11), personality trait: extroversion (21.18 ± 8.96), agreeableness (28.01 ± 7.48), conscientiousness (25.20 ± 7.06), neuroticism (19.29 ± 8.94) and openness (27.36 ± 7.81). Other characteristics of the participants are summarized in Table 1.

**Table 1:** Sociodemographic and other characteristics of the participants (*N* = 296)

Variable	N (%)
**Gender**	
Male	130 (43.9%)
Female	166 (56.1%)
**Governorate**	
Beirut	28 (9.5%)
Mount Lebanon	124 (41.9%)
North	74 (25.0%)
South	37 (12.5%)
Bekaa	33 (11.1%)
**Monthly income**	
Low (< 1000 USD)	21 (7.1%)
Intermediate (1000–2000 USD)	133 (44.9%)
High (> 2000 USD)	142 (48.0%)
**Mother’s education level**	
Primary/complementary	17 (5.7%)
High school	52 (17.6%)
Undergraduate diploma	75 (25.3%)
Graduate diploma	109 (36.8%)
Postgraduate diploma	43 (14.5%)
**Father’s education level**	
Primary/complementary	38 (12.8%)
High school	30 (10.1%)
Undergraduate diploma	51 (17.2%)
Graduate diploma	99 (33.4%)
Postgraduate diploma	78 (26.4%)
**University**	
American University of Beirut	20 (6.8%)
Beirut Arab University	13 (4.4%)
Holy Spirit University of Kaslik	133 (44.9%)
Lebanese American University	17 (5.7%)
Lebanese University	80 (27.0%)
Saint Joseph University	14 (4.7%)
University of Balamand	19 (6.4%)

### Bivariate analysis

Males vs females, having chronic pain compared to not, originating from South Lebanon compared to other governorates, having an intermediate income compared to other categories, those whose mothers had a primary/complementary education level and those whose fathers had an undergraduate diploma vs all other categories had higher mean rationale style scores. Those fathers, who had a postgraduate diploma, had a higher mean intuitive style scores compared to all other education levels. Those who have chronic pain compared to not and living in South Lebanon compared to other governorates had higher dependent style scores. Those who have chronic pain compared to not, those who take medications for a mental illness whose mothers had a primary/complementary education level vs all other categories and those whose fathers had a postgraduate diploma vs all other categories had higher spontaneous style scores (Table 2).

**Table 2:** Bivariate analysis of factors associated with the decision-making subscales scores

Variable	Rationale style	Intuitive style	Dependent style	Spontaneous style	Avoidant style
**Gender**					
Male	13.62 ± 3.38	15.63 ± 3.98	15.54 ± 4.12	13.69 ± 4.28	11.79 ± 4.27
Female	12.64 ± 2.94	16.37 ± 3.89	15.53 ± 4.39	13.38 ± 4.17	12.95 ± 3.93
p	**0.006**	0.111	0.785	0.578	0.107
**Chronic pain**					
No	13.28 ± 3.06	16.04 ± 3.88	15.88 ± 4.10	13.70 ± 4.23	12.12 ± 3.70
Yes	11.56 ± 3.58	16.06 ± 4.44	13.06 ± 4.68	12.17 ± 3.95	14.75 ± 15.90
p	**0.011**	0.962	**0.001**	**0.011**	**0.036**
**Medications intake for mental illness**					
No	13.26 ± 2.99	15.93 ± 3.84	15.52 ± 4.18	13.51 ± 4.15	11.95 ± 3.53
Yes	11.58 ± 4.09	16.94 ± 4.63	15.61 ± 4.95	13.58 ± 4.79	16.30 ± 6.04
p	0.129	0.170	0.359	0.885	**< 0.001**
**Governorate**					
Beirut	11.14 ± 3.61	18.11 ± 4.69	12.86 ± 5.08	14.89 ± 4.00	12.39 ± 5.47
Mount Lebanon	13.11 ± 2.46	16.15 ± 3.11	15.90 ± 3.84	13.60 ± 3.64	12.44 ± 3.03
North	12.70 ± 3.44	16.23 ± 4.12	15.47 ± 4.12	13.80 ± 4.22	13.12 ± 4.35
South	15.14 ± 3.20	15.14 ± 3.65	16.68 ± 3.88	11.89 ± 4.45	11.65 ± 4.63
Bekaa	13.06 ± 3.38	14.48 ± 5.15	15.30 ± 5.02	13.21 ± 5.71	11.85 ± 5.08
p	**0.001**	0.065	**0.027**	0.068	0.177
**Monthly income**					
Low (< 1000 $)	12.48 ± **2.23**	16.81 ± 3.82	15.10 ± 3.65	13.33 ± 3.29	12.71 ± 3.69
Intermediate (1000–2000 $)	13.64 ± 2.72	15.62 ± 3.59	16.17 ± 3.35	13.41 ± 4.27	12.85 ± 3.97
High (> 2000 $)	12.63 ± 3.58	16.33 ± 4.25	15.01 ± 5.00	13.64 ± 4.31	12.01 ± 4.28
p	**0.017**	0.669	0.196	0.806	0.146
**Mother’s educational level**					
Primary/ complementary	13.88 ± 1.22	14.88 ± 2.62	15.76 ± 2.93	13.29 ± 2.62	13.59 ± 2.98
High school	13.83 ± 2.74	15.77 ± 3.53	15.13 ± 3.44	13.23 ± 3.66	13.06 ± 3.40
Undergraduate diploma	12.23 ± 3.73	16.15 ± 5.22	15.45 ± 5.61	14.17 ± 5.28	10.63 ± 4.24
Graduate diploma	12.86 ± 3.19	16.17 ± 3.54	16.10 ± 3.89	13.25 ± 4.17	12.76 ± 4.19
Postgraduate diploma	13.84 ± 2.70	16.33 ± 3.22	14.63 ± 3.72	13.49 ± 3.35	13.58 ± 4.01
p	**0.014**	0.604	0.530	0.757	**< 0.001**
**Father’s education level**					
Primary/ complementary	13.84 ± 1.37	15.08 ± 2.83	15.53 ± 2.94	13.79 ± 2.52	12.68 ± 2.65
High school	13.73 ± 1.41	14.57 ± 2.94	16.40 ± 2.13	13.37 ± 3.54	12.67 ± 2.73
Undergraduate diploma	14.04 ± 3.80	15.43 ± 5.15	15.90 ± 5.15	13.41 ± 5.70	11.49 ± 4.58
Graduate diploma	12.25 ± 3.39	16.46 ± 4.28	15.31 ± 4.77	13.96 ± 4.50	12.05 ± 4.60
Postgraduate diploma	12.85 ± 3.29	16.95 ± 3.05	15.24 ± 4.13	12.95 ± 3.60	13.35 ± 4.04
p	**0.002**	**0.008**	0.585	0.697	**0.019**

Higher agreeableness and conscientiousness scores were significantly associated with higher rational style scores, whereas higher extroversion and neuroticism scores were significantly associated with lower rational style scores. Higher extroversion, openness and emotional intelligence scores were significantly associated with higher intuitive scores, whereas higher agreeableness, conscientiousness and neuroticism scores were significantly associated with lower intuitive style scores. Higher agreeableness and conscientiousness were associated with higher dependent style scores, whereas higher openness and emotional intelligence scores were significantly associated with lower dependent styles scores. Higher agreeableness, conscientiousness, neuroticism, and emotional intelligence scores were significantly associated with lower spontaneous style scores. Finally, higher extroversion, neuroticism and emotional intelligence scores were significantly associated with lower avoidant style scores (Table 3).

**Table 3:** Bivariate analysis of continuous variables associated with the decision-making subscales scores

Variable	Rationale style	Intuitive style	Dependent style	Spontaneous style	Avoidant style
Extroversion	−0.137^c^	0.146^c^	−0.109	0.043	−0.126^c^
Agreeableness	0.115^c^	−0.195^b^	0.342^a^	−0.195^b^	0.091
Conscientiousness	0.227^a^	−0.107	0.160^b^	−0.272^a^	0.028
Neuroticism	−0.157^b^	−0.033	− 0.063	−0.177^b^	− 0.237^a^
Openness	0.048	0.154^b^	−0.225^a^	−0.113	0.065
Emotional intelligence total score	0.029	0.167^b^	−0.165^b^	−0.155^b^	− 0.183^b^
Age	0.086	0.035	−0.085	0.110	0.033

^a^*p* < 0.001; ^b^*p* < 0.01; ^c^*p* < 0.05

Post hoc analysis: rationale style: governorate (Beirut vs Mount Lebanon *p* = 0.022; Beirut vs South *p* < 0.001; Mount Lebanon vs South *p* = 0.004; South vs North *p* = 0.001; South vs Bekaa *p* = 0.047); monthly income (intermediate vs high *p* = 0.024); mother’s educational level (high school vs undergraduate diploma *p* = 0.048); father’s education level (undergraduate vs graduate diploma p = 0.01).

Intuitive style: father’s education level (high school vs postgraduate diploma *p* = 0.046).

Dependent style: governorate (Beirut vs Mount Lebanon *p* = 0.006; Beirut vs South *p* = 0.003);

Avoidant style: mother’s educational level (high school vs undergraduate diploma *p* = 0.008; undergraduate vs graduate diploma *p* = 0.004; undergraduate vs postgraduate diploma *p* = 0.001).

### Mediation analysis

Mediation analysis was run to check if emotional intelligence would have a mediating role between each personality trait and each decision-making style, after adjusting overall covariates that showed a *p* < 0.05 with each decision-making style in the bivariate analysis.

#### Rational decision-making style (Table 4, model 1)

Higher extroversion was significantly associated with higher EI, b = 0.91, 95% BCa CI [0.60, 1.23], t = 5.71, *p* < 0.001 (R2 = 0.31). Higher extroversion was significantly associated with lower rational decision-making even with EI in the model, b = − 0.06, 95% BCa CI [− 0.11, − 0.02], t = − 2.81, *p* = 0.003; EI was not significantly associated with rational decision-making, b = 0.02, 95% BCa CI [− 0.0003, 0.03], t = 1.93, *p* = 0.054 (R2 = 0.29). When EI was not in the model, higher extroversion was significantly associated with lower rational decision-making, b = − 0.05, 95% BCa CI [− 0.09, − 0.01], t = − 2.43, *p* = 0.015 (R2 = 0.28). The mediating effect of EI was 21.22%.

**Table 4:** Mediation analysis

**Model 1: Rational decision-making style.**																
	Effect of personality trait on EI					Effect of personality trait and EI on rational decision-making style					Direct effect of personality trait on rational decision-making style					Mediating effect of EI
	Beta	95% BCa CI		t	p	Beta	95% BCa CI		t	p	Beta	95% BCa CI		t	p	
Extroversion	0.91	0.60	1.23	5.71	**< 0.001**	−0.06	− 0.11	− 0.02	− 2.81	**0.003**	− 0.05	− 0.09	− 0.01	− 2.43	**0.015**	21.22%
EI						0.02	−0.0003	0.03	1.93	0.054						
Agreeableness	−0.05	−0.40	0.31	−0.26	0.798	0.07	0.02	0.11	2.89	**0.004**	0.07	0.02	0.11	2.86	**0.004**	0.10%
EI						0.01	−0.0003	0.03	1.92	0.055						
Conscientiousness	1.40	1.04	1.76	7.62	< 0.001	0.09	0.04	0.14	3.55	**< 0.001**	0.11	0.07	0.16	4.76	**< 0.001**	22.47%
EI						0.01	−0.0003	0.03	1.93	0.055						
Neuroticism	−0.50	− 0.80	− 0.20	−3.26	**0.001**	− 0.09	− 0.05	0.03	− 0.43	0.668	− 0.02	−0.06	0.02	−0.81	0.418	–
EI						0.01	−0.0003	0.03	1.93	0.055						
**Model 2: Intuitive decision-making style.**																
	Effect of personality trait on EI					Effect of personality trait and EI on intuitive decision-making style					Direct effect of personality trait on intuitive decision-making style					Mediating effect of EI
	Beta	95% BCa CI		t	p	Beta	95% BCa CI		t	p	Beta	95% BCa CI		t	p	
Extroversion	0.86	0.59	1.13	6.28	**< 0.001**	0.05	0.002	0.11	2.03	**0.043**	0.08	0.03	0.13	3.21	**0.001**	49.82%
EI						0.03	0.01	0.05	2.91	**0.003**						
Agreeableness	−0.33	−0.65	− 0.02	−2.06	**0.039**	−0.15	−0.21	− 0.10	−2.16	**< 0.001**	− 0.17	− 0.22	−0.11	−5.48	**< 0.001**	6.80%
EI						0.03	0.01	0.05	2.91	**0.004**						
Conscientiousness	1.18	0.85	1.51	7.06	**< 0.001**	−0.10	− 0.16	− 0.03	−2.95	**0.003**	− 0.06	− 0.12	0.0004	−1.95	0.051	38%
EI						0.03	0.01	0.05	2.91	**0.004**						
Openness to experience	1.44	1.13	1.75	9.11	**< 0.001**	0.08	0.01	0.14	2.38	**0.017**	0.12	0.06	0.18	4.22	**< 0.001**	57.93%
EI						0.03	0.01	0.05	2.91	**0.004**						
**Model 3: Dependent decision-making style.**																
	Effect of personality trait on EI					Effect of personality trait and EI on dependent decision-making style					Direct effect of personality trait on dependent decision-making style					Mediating effect of EI
	Beta	95% BCa CI		t	p	Beta	95% BCa CI		t	p	Beta	95% BCa CI		t	p	
Agreeableness	−0.15	−0.49	0.17	7.73	**< 0.001**	0.29	0.23	0.34	10.51	**< 0.001**	0.29	0.24	0.35	10.44	**< 0.001**	2.38%
EI						−0.04	−0.06	− 0.02	−4.50	**< 0.001**						
Conscientiousness	1.04	0.69	1.38	5.93	**< 0.001**	0.15	0.09	0.20	4.88	**< 0.001**	0.10	0.04	0.16	3.49	**< 0.001**	30.25%
EI						−0.04	−0.06	− 0.02	−4.50	**< 0.001**						
Openness to experience	1.37	1.05	1.69	8.41	**< 0.001**	− 0.13	− 0.19	− 0.08	−4.55	**< 0.001**	− 0.19	− 0.24	− 0.14	−7.06	**< 0.001**	43.69%
EI						−0.04	− 0.19	− 0.08	−4.50	**< 0.001**						
**Model 4: Spontaneous decision-making style.**																
	Effect of personality trait on EI					Effect of personality trait and EI on spontaneous decision-making style					Direct effect of personality trait on spontaneous decision-making style					Mediating effect of EI
	Beta	95% BCa CI		t	p	Beta	95% BCa CI		t	p	Beta	95% BCa CI		t	p	
Agreeableness	0.17	−0.19	0.53	0.91	0.364	−0.10	− 0.16	− 0.03	−3.07	**0.002**	− 0.10	− 0.16	−0.04	−3.11	**0.002**	1.25%
EI						−0.01	−0.03	0.01	−0.71	0.476						
Conscientiousness	1.26	0.88	1.64	6.56	**< 0.001**	−0.16	− 0.23	− 0.09	−4.51	**< 0.001**	− 0.17	− 0.23	− 0.10	−5.11	**< 0.001**	5.64%
EI						−0.01	− 0.03	0.01	− 0.71	0.476						
Neuroticism	−0.22	−0.53	0.08	−1.43	0.153	−0.11	−0.16	− 0.06	−4.05	**< 0.001**	− 0.11	− 0.16	− 0.05	− 4.01	**< 0.001**	1.49%
EI						−0.01	− 0.03	0.01	− 0.71	0.476						
**Model 5: Avoidant decision-making style.**																
	Effect of personality trait on EI					Effect of personality trait and EI on avoidant decision-making style					Direct effect of personality trait on avoidant decision-making style					Mediating effect of EI
	Beta	95% BCa CI		t	p	Beta	95% BCa CI		t	p	Beta	95% BCa CI		t	p	
Extroversion	0.88	0.54	1.21	5.18	**< 0.001**	−0.01	−0.06	0.05	−0.27	0.790	−0.05	− 0.10	0.08	−1.69	0.092	–
EI						−0.04	−0.06	0.03	−4.79	**< 0.001**						
Neuroticism	−0.59	−0.91	−0.27	−3.60	**< 0.001**	− 0.03	− 0.09	0.02	−1.34	0.182	− 0.09	− 0.06	0.04	− 0.33	0.739	–
EI						−0.04	− 0.06	− 0.03	−4.79	**< 0.001**						

Higher agreeableness was not significantly associated with EI, b = − 0.05, 95% BCa CI [− 0.40, 0.31], t = − 0.26, *p* = 0.798 (R2 = 0.31). Higher agreeableness was significantly associated with higher rational decision-making style even with EI in the model, b = 0.07, 95% BCa CI [0.02, 0.11], t = 2.89, *p* = 0.004; EI was not significantly associated with the rational decision-making, b = 0.01, 95% BCa CI [− 0.0003, 0.03], t = 1.92, *p* = 0.055 (R2 = 0.29). When EI was not in the model, higher agreeableness was significantly associated with higher rational decision-making, b = 0.07, 95% BCa CI [0.02, 0.11], t = 2.86, p = 0.004 (R2 = 0.28). The mediating effect of EI was 0.10%.

Higher conscientiousness was significantly associated with higher EI, b = 1.40, 95% BCa CI [1.04, 1.76], t = 7.62, *p* < 0.001 (R2 = 0.31). Higher conscientiousness was significantly associated with the rational decision-making style even with EI in the model, b = 0.09, 95% BCa CI [0.04, 0.14], t = 3.55, p < 0.001; EI was not significantly associated with the rational decision-making, b = 0.01, 95% BCa CI [− 0.0003, 0.03], t = 1.93, *p* = 0.055 (R2 = 0.29). When EI was not in the model, conscientiousness was significantly associated with the rational decision-making style, b = 0.11, 95% BCa CI [0.07, 0.16], t = 4.76, p < 0.001 (R2 = 0.28). The mediating effect of EI was 22.47%.

Higher neuroticism was significantly associated with lower EI, b = − 0.50, 95% BCa CI [− 0.80, − 0.20], t = − 3.26, *p* = 0.001 (R2 = 0.31). Neuroticism was not significantly associated with rational decision-making style with EI in the model, b = − 0.09, 95% BCa CI [− 0.05, 0.03], t = − 0.43, *p* = 0.668; EI was not significantly associated with rational decision-making, b = 0.01, 95% BCa CI [− 0.0003, 0.03], t = 1.93, *p* = 0.055 (R2 = 0.29). When EI was not in the model, neuroticism was not significantly associated with the rational decision-making style, b = − 0.02, 95% BCa CI [− 0.06, 0.02], t = − 0.81, *p* = 0.418 (R2 = 0.28).

No calculations were done for the openness to experience personality traits since it was not significantly associated with the rational decision-making style in the bivariate analysis.

#### Intuitive decision-making style (Table 4, model 2)

Higher extroversion was significantly associated with higher EI, b = 0.86, 95% BCa CI [0.59, 1.13], t = 6.28, *p* < 0.001 (R2 = 0.41). Higher extroversion was significantly associated with higher intuitive decision-making even with EI in the model, b = 0.05, 95% BCa CI [0.002, 0.11], t = 2.03, *p* = 0.043; EI was significantly associated with intuitive decision-making style, b = 0.03, 95% BCa CI [0.01, 0.05], t = 2.91, *p* = 0.003 (R2 = 0.21). When EI was not in the model, higher extroversion was significantly associated with higher intuitive decision-making, b = 0.08, 95% BCa CI [0.03, 0.13], t = 3.21, *p* = 0.001 (R2 = 0.18). The mediating effect of EI was 49.82%.

Higher agreeableness was significantly associated with EI, b = − 0.33, 95% BCa CI [− 0.65, − 0.02], t = − 2.06, *p* = 0.039 (R2 = 0.41). Higher agreeableness was significantly associated with lower intuitive decision-making style even with EI in the model, b = − 0.15, 95% BCa CI [− 0.21, − 0.10], t = − 5.16, *p* < 0.001; higher EI was significantly associated with higher intuitive decision-making, b = 0.03, 95% BCa CI [0.01, 0.05], t = 2.91, *p* = 0.004 (R2 = 0.21). When EI was not in the model, higher agreeableness was significantly associated with lower intuitive decision-making, b = − 0.17, 95% BCa CI [− 0.22, − 0.11], t = − 5.48, p < 0.001 (R2 = 0.18). The mediating effect of EI was 6.80%.

Higher conscientiousness was significantly associated with higher EI, b = 1.18, 95% BCa CI [0.85, 1.51], t = 7.06, p < 0.001 (R2 = 0.41). Higher conscientiousness was significantly associated with lower intuitive decision-making style even with EI in the model, b = − 0.10, 95% BCa CI [− 0.16, − 0.03], t = − 2.95, *p* = 0.003; higher EI was also significantly associated with higher intuitive decision-making, b = 0.03, 95% BCa CI [0.01, 0.05], t = 2.91, *p* = 0.004 (R2 = 0.21). When EI was not in the model, conscientiousness was not significantly associated with the intuitive decision-making style, b = − 0.06, 95% BCa CI [− 0.12, 0.0004], t = − 1.95, *p* = 0.051 (R2 = 0.18). The mediating effect of EI was 38%.

Higher openness to experience was significantly associated with higher EI, b = 1.44, 95% BCa CI [1.13, 1.75], t = 9.11, *p* < 0.001 (R2 = 0.41). Higher openness to experience was significantly associated with higher intuitive decision-making style with EI in the model, b = 0.08, 95% BCa CI [0.01, 0.14], t = 2.38, *p* = 0.017; higher EI was also significantly associated with intuitive decision-making style, b = 0.03, 95% BCa CI [0.01, 0.05], t = 2.91, *p* = 0.004 (R2 = 0.21). When EI was not in the model, higher openness to experience was significantly associated with intuitive decision-making style, b = 0.12, 95% BCa CI [0.06, 0.18], t = 4.22, *p* < 0.001 (R2 = 0.18). The mediating effect of EI was 57.93%.

No calculations were done for neuroticism personality trait since it was not significantly associated with the intuitive decision-making style in the bivariate analysis.

#### Dependent decision-making style (Table 4, model 3)

Agreeableness was not significantly associated with EI, b = − 0.15, 95% BCa CI [− 0.49, 0.17], t = − 0.94, *p* = 0.345 (R2 = 0.32). Higher agreeableness was significantly associated with higher dependent decision-making style even with EI in the model, b = 0.29, 95% BCa CI [0.23, 0.34], t = 10.51, *p* < 0.001; higher EI was significantly associated with lower dependent decision-making, b = − 0.04, 95% BCa CI [− 0.06, − 0.02], t = − 4.50, *p* < 0.001 (R2 = 0.40). When EI was not in the model, higher agreeableness was significantly associated with higher dependent decision-making, b = 0.29, 95% BCa CI [0.24, 0.35], t = 10.44, *p* < 0.001 (R2 = 0.18). The mediating effect of EI was 2.38%.

Higher conscientiousness was significantly associated with higher EI, b = 1.04, 95% BCa CI [0.69, 1.38], t = 5.93, *p* < 0.001 (R2 = 0.32). Higher conscientiousness was significantly associated with higher dependent decision-making style even with EI in the model, b = 0.15, 95% BCa CI [0.09, 0.20], t = 4.88, *p* < 0.001; higher EI was also significantly associated with lower dependent decision-making, b = − 0.04, 95% BCa CI [− 0.06, − 0.02], t = − 4.50, *p* < 0.001 (R2 = 0.40). When EI was not in the model, higher conscientiousness was significantly associated with a higher dependent decision-making style, b = 0.10, 95% BCa CI [0.04, 0.16], t = 3.49, *p* < 0.001 (R2 = 0.36). The mediating effect of EI was 30.25%.

Higher openness to experience was significantly associated with higher EI, b = 1.37, 95% BCa CI [1.05, 1.69], t = 8.41, *p* < 0.001 (R2 = 0.32). Higher openness to experience was significantly associated with lower dependent decision-making style even with EI in the model, b = − 0.13, 95% BCa CI [− 0.19, − 0.08], t = − 4.55, p < 0.001; higher EI was also significantly associated with dependent decision-making style, b = − 0.04, 95% BCa CI [− 0.19, − 0.08], t = − 4.50, p < 0.001 (R2 = 0.40). When EI was not in the model, higher openness to experience was significantly associated with lower dependent decision-making style, b = − 0.19, 95% BCa CI [− 0.24, − 0.14], t = − 7.06, p < 0.001 (R2 = 0.36). The mediating effect of EI was 43.69%.

No calculations were done for neuroticism and extroversion personality traits since they were not significantly associated with the dependent decision-making style in the bivariate analysis.

#### Spontaneous decision-making style (Table 4, model 4)

Agreeableness was not significantly associated with EI, b = 0.17, 95% BCa CI [− 0.19, 0.53], t = 0.91, *p* = 0.364 (R2 = 0.17). Higher agreeableness was significantly associated with lower spontaneous decision-making style even with EI in the model, b = − 0.10, 95% BCa CI [− 0.16, − 0.03], t = − 3.07, *p* = 0.002; EI was not significantly associated with spontaneous decision-making, b = − 0.01, 95% BCa CI [− 0.03, 0.01], t = − 0.71, *p* = 0.476 (R2 = 0.15). When EI was not in the model, higher agreeableness was significantly associated with lower spontaneous decision-making, b = − 0.10, 95% BCa CI [− 0.16, − 0.04], t = − 3.11, p = 0.002 (R2 = 0.15). The mediating effect of EI was 1.25%.

Higher conscientiousness was significantly associated with higher EI, b = 1.26, 95% BCa CI [0.88, 1.64], t = 6.56, *p* < 0.001 (R2 = 0.17). Higher conscientiousness was significantly associated with lower spontaneous decision-making style even with EI in the model, b = − 0.16, 95% BCa CI [− 0.23, − 0.09], t = − 4.51, *p* < 0.001; EI was not significantly associated with spontaneous decision-making style, b = − 0.01, 95% BCa CI [− 0.03, 0.01], t = − 0.71, *p* = 0.476 (R2 = 0.15). When EI was not in the model, higher conscientiousness was significantly associated with lower spontaneous decision-making style, b = − 0.17, 95% BCa CI [− 0.23, − 0.10], t = − 5.11, *p* < 0.001 (R2 = 0.15). The mediating effect of EI was 5.64%.

Neuroticism was not significantly associated with EI, b = − 0.22, 95% BCa CI [− 0.53, 0.08], t = − 1.43, *p* = 0.153 (R2 = 0.17). Higher neuroticism was significantly associated with lower spontaneous decision-making style even with EI in the model, b = − 0.11, 95% BCa CI [− 0.16, − 0.06], t = − 4.05, *p* < 0.001; EI was not significantly associated with spontaneous decision-making style, b = − 0.01, 95% BCa CI [− 0.03, 0.01], t = − 0.71, p = 0.476 (R2 = 0.15). When EI was not in the model, higher neuroticism was significantly associated with lower spontaneous decision-making style, b = − 0.11, 95% BCa CI [− 0.16, − 0.05], t = − 4.01, *p* < 0.001 (R2 = 0.15). The mediating effect of EI was 1.49%.

No calculations were done for openness to experience and extroversion personality traits since they were not significantly associated with the spontaneous decision-making style in the bivariate analysis**.**

#### Avoidant decision-making style (Table 4, model 5)

Higher extroversion was significantly associated with higher EI, b = 0.88, 95% BCa CI [0.54, 1.21], t = 5.18, *p* < 0.001 (R2 = 0.15). Extroversion was not significantly associated with avoidant decision-making style even with EI in the model, b = − 0.01, 95% BCa CI [− 0.06, 0.05], t = − 0.27, *p* = 0.790; higher EI was significantly associated with avoidant decision-making style, b = − 0.04, 95% BCa CI [− 0.06, 0.03], t = − 4.79, *p* < 0.001 (R2 = 0.25). When EI was not in the model, extroversion was not significantly associated with avoidant decision-making style, b = − 0.05, 95% BCa CI [− 0.1, 0.08], t = − 1.69, *p* = 0.092 (R2 = 0.19).

Higher neuroticism was significantly associated with lower EI, b = − 0.59, 95% BCa CI [− 0.91, − 0.27], t = − 3.60, p < 0.001 (R2 = 0.15). Neuroticism was not significantly associated with avoidant decision-making style even with EI in the model, b = − 0.03, 95% BCa CI [− 0.09, 0.02], t = − 1.34, *p* = 0.182; higher EI was significantly associated with lower avoidant decision-making style, b = − 0.04, 95% BCa CI [− 0.06, − 0.03], t = − 4.79, p < 0.001 (R2 = 0.25). When EI was not in the model, neuroticism was not significantly associated with avoidant decision-making style, b = − 0.09, 95% BCa CI [− 0.06, 0.04], t = − 0.33, *p* = 0.739 (R2 = 0.19).

No calculations were done for openness to experience, agreeableness, and conscientiousness personality traits since they were not significantly associated with the avoidant decision-making style in the bivariate analysis.

## Discussion

This study examined the relationship between personality traits and decision-making styles, and the mediation role of emotional intelligence in a sample of general medicine students from different medical schools in Lebanon.

Agreeableness is characterized by cooperation, morality, sympathy, low self-confidence, high levels of trust in others and agreeable individuals tend to be happy and satisfied because of their close interrelationships [19, 20]. Likewise, dependent decision-making style is characterized by extreme dependence on others when it comes to making decisions [1]. Our study confirmed this relationship similarly to Wood (2012) [41] and Bayram and Aydemir (2017) [26] findings of a positive relationship between dependent decision-making style and agreeableness personality trait and a negative correlation between this same personality trait and spontaneous decision-making style. In fact, this negative correlation can be explained by the reliance and trust accorded by agreeable individuals to their surroundings, making them highly influenced by others opinions when it comes to making a decision; hence, avoiding making rapid and snap decisions on the spur of the moment (i.e. spontaneous decision-making style); in order to explore the point of view of their surrounding before deciding on their own.

Conscientiousness is characterized by competence, hard work, self-discipline, organization, strive for achievement, and goal orientation [20]. Besides, conscientious individuals have a high level of deliberation making them capable of analyzing the pros and cons of a given situation [21]. Similarly, rational decision-makers strive for achievements by searching for information and logically evaluating alternatives before making decisions; making them high achievement-oriented [20, 42]. This positive relationship between rational decision-making style and conscientiousness was established by Nygren and White (2005) [43] and Bajwa et al. (2016) [25]; thus, solidifying our current findings. Furthermore, we found that conscientiousness was positively associated with dependent decision-making; this relationship was not described in previous literature to our knowledge and remained statistically significant after adding EI to the analysis model. This relationship may be explained by the fact that conscientious individuals tend to take into consideration the opinions of their surrounding in their efforts to analyze the pros and cons of a situation. Further investigations in similar populations should be conducted in order to confirm this association. Moreover, we found a positive relationship between conscientiousness and intuitive decision-making that lost significance when EI was removed from the model. Thus, solidifying evidence of the mediating role played by EI between personality trait and decision-making style with an estimated mediation effect of 38%.

Extroversion is characterized by higher levels of self-confidence, positive emotions, enthusiasm, energy, excitement seeking, and social interactions. Similarly, intuitive decision-making is highly influenced by emotions and instinct. The positive relationship between extroversion and intuitive decision-making style was supported by Wood (2012) [41], Riaz et al. (2012) [24] and Narooi and Karazee (2015) [23] findings and by our present study.

Neuroticism is characterized by anxiety, anger, self-consciousness, and vulnerability [20]. High neurotic individuals have higher levels of negative affect, depression, are easily irritated, and more likely to turn to inappropriate coping responses, such as interpersonal hostility [22]. Our study results showed a negative relationship between neuroticism and spontaneous decision-making style.

Openness to experience individuals are creative, imaginative, intellectually curious, impulsive and original, open to new experiences and ideas [19, 20]. One important characteristic of intuitive decision-making style is tolerance for ambiguity and the ability to picture the problem and its potential solution [44]. The positive relationship between openness to experience and intuitive decision-making style was established by Riaz and Batool (2012) [24] and came in concordance with our study findings. Additionally, our results suggest that openness personality trait is negatively associated with dependent decision-making style similar to previous findings [23]. Openness to experience individuals are impulsive and continuously seek intellectual pursuits and new experiences; hence, they tend to depend to a lesser extent on others’ opinions when making decisions since they consider the decision-making process a way to uncover new experiences and opportunities.

Our study results showed that EI had a significant positive effect on intuitive decision-making style. Intuition can be regarded as an interplay between cognitive and affective processes highly influenced by tactic knowledge [45]; hence, intuitive decision-making style is the result of personal and environmental awareness [46–48] in which individuals rely on the overall context without much concentration on details. In other words, they depend on premonitions, instinct, and predications of possibilities focusing on designing the overall plan [49] and take responsibility for their decisions [46]. Our study finding supports the results of Khan and al. (2016) who concluded that EI and intuitive decision-making had a positive relationship [35]. On the other hand, our study showed a negative relationship between EI and avoidant and dependent decision-making styles. Avoidant decision-making style is defined as a continuous attempt to avoid decision-making when possible [1] since they find it difficult to act upon their intentions and lack personal and environmental awareness [50]. Similarly to our findings, Khan and al. (2016) found that avoidant style is negatively influenced by EI [35]. The dependent decision-making style can be regarded as requiring support, advice, and guidance from others when making decisions. In other words, it can be described as an avoidance of responsibility and adherence to cultural norms; thus, dependent decision-makers tend to be less influenced by their EI in the decision-making process. Our conclusion supports Avsec’s (2012) findings [51] on the negative relationship between EI and dependent decision-making style.

### Practical implications

The present study helps in determining which sort of decision is made by which type of people. This study also represents a valuable contribution to the Lebanese medical society in order to implement such variables in the selection methods of future physicians thus recruiting individuals with positively evaluated decision-making styles and higher levels of emotional intelligence; implying better communication skills and positively impacting patients’ experience. Also, the present study may serve as a valuable tool for the medical school administration to develop targeted measures to improve students’ interpersonal skills.

### Limitations

Even though the current study is an important tool in order to understand the complex relationship between personality traits, decision-making styles and emotional intelligence among medical students; however, it still carries some limitations. This study is a descriptive cross-sectional study thus having a lower internal validity in comparison with experimental studies. The Scott and Bruce General Decision-Making Style Inventory has been widely used internationally for assessing decision-making styles since 1995 but has not been previously validated in the Lebanese population. In addition, the questionnaire was only available in English taking into consideration the mandatory good English knowledge in all the Lebanese medical schools; however, translation, and cross-language validation should be conducted in other categories of Lebanese population. Furthermore, self-reported measures were employed in the present research where participants self-reported themselves on personality types, decision-making styles and emotional intelligence. Although, all used scales are intended to be self-administered; however, this caries risk of common method variance; hence, cross-ratings may be employed in the future researches in order to limit this variance.

## Conclusion

The results suggest that EI showed a significant positive effect on intuitive decision-making style and a negative effect on avoidant and dependent decision-making styles. In addition, our study showed a positive relationship between agreeableness and dependent decision-making style and a negative correlation with spontaneous decision-making style. Furthermore, conscientiousness had a positive relationship with rational and dependent decision-making style and extroversion showed a positive relationship with intuitive decision-making style. Neuroticism had a negative relationship with spontaneous style and openness to experience showed a positive relationship with intuitive decision-making style and a negative relationship with dependent style. Additionally, our study underlined the role of emotional intelligence as a mediation factor between personality traits and decision-making styles namely openness to experience, extroversion, and conscientiousness personality traits with intuitive decision-making style. Personality traits are universal [20]; beginning in adulthood and remaining stable with time [52]. Comparably, decision-making styles are stable across situations [1]. The present findings further solidify a previously established relationship between personality traits and decision-making and describes the effect of emotional intelligence on this relationship.

## Data Availability

All data generated or analyzed during this study are not publicly available to maintain the privacy of the individuals’ identities. The dataset supporting the conclusions is available upon request to the corresponding author.
